# Quartz Light (Ultra Violet Radiation)

**Published:** 1923-04

**Authors:** G. W. Ross

**Affiliations:** Toronto, Canada


### QUARTZ LIGHT (ULTRA VIOLET RADIATION)

---

BY DR. G. W. ROSS, TORONTO, CANADA

---

Light therapy or heliotherapy is among the oldest known therapeutic measures. Its value is vouched for in the writing of Herodotus (B. C. ——) and of Hippocrates. Solaria were common in the homes of the wealthier of the ancient Greeks and Romans. But only about 1800 was the method of sun therapy revived and then by the French. Throughout the nineteenth century in France, Germany and Switzerland we find efforts made to interest the profession in the method with but little success. However, in 1899, Finsen's scientific studies on Sunlight and especially on the violet and ultra violet rays attracted widespread attention in the medical world. Among other things he demonstrated that the red end of the solar spectrum and beyond (the infra-red) were responsible for the heat rays, while the violet end and beyond were actinic and largely responsible for any photo-chemical reactions. His researches further demonstrated that the red-blood corpuscles absorbed the healing or chemical rays of sunlight and also of the carbon arc light, and so it was that he developed the lamp that bears his name. Its effectiveness in Lupus Vulgaris, for example, was due first to the production of Ultra Violet rays and secondly to their introduction into tuberculosis foci by dehaematization of adjoining tissues by means of pressure.

While the Finsen and the less cumbersome Finsen-Rezn lamps represented distinct advances in photo-therapy partly by demonstrating their clinical value, especially in Lupus Vulgaris (hitherto an almost incurable malady), they did so especially through the research that was stimulated into the physical properties of the various electro-magnetic waves emitted by the sun and also those derived from many other sources.

The final product of these investigations, the so-called Mercury Quartz Lamp, represents the work of many investigators; but we owe it chiefly to ARONS, who in 1892 discovered that a container with Mercury vapor, if electrified, emitted a bluish light without the red or orange rays of the spectrum; and later HERAEUS who succeeded in fusing quartz crystals. This last discovery was fundamental for two reasons, first because it permitted the manufacture of a vacuum tube to contain the mercury, capable of retaining its consistency at a very high temperature (namely, 5000° F.) and second because unlike glass, Quartz is permeable to the Ultra Violet rays. In 1904 KROMAYER applied these discoveries to the construction of the lamp that bears his name and somewhat later NAGELSCHMIDT devised the air-cooled lamp (so-called Alpine Lamp) which permitted of larger radiations.

Throughout this period of less than ten years one discovers on the part of these investigators, and many others not mentioned, a determination to extract from artificial sources of energy the "healing rays of the sun"; and there can be little doubt that the final result has been successful; but before proceeding to discuss the two forms of so-called lamps in common use for this purpose, certain scientific facts are worthy at least of a passing notice if we are to attune our minds to a proper understanding of the subject.

When sunlight traverses a prism it is split up into many rays—those that are visible, the seven colors of the rainbow, and those that are invisible. These rays, visible and invisible, according to physicists, are electro-magnetic vibrations and their various properties whether of color, the production of heat or of chemical reactions depend entirely upon their wave-lengths.

The wave-length of the red in the spectrum is about one-half inch, that of the violet about one-quarter inch; to the left or below the visible red are the infra-red of variable length, up to the long Hertzian waves of wireless fame perhaps a mile long; to the right of the violet are the Ultra Violet rays again of varying length from those nearest the visible violet emitted by the Mercury Quartz Lamp to the X-rays and the Gamma rays of radium estimated at quintillionths of an inch.

Modern physical research has made commonplace some of the most extraordinary discoveries of all time, namely, wireless telegraphy and telephony, X-rays and radium. These things we all know and if we will but accept the broader conception that each represents an electro-magnetic force exhibiting itself peculiarly, because of particular wave-lengths, it is not difficult to allow our minds to scan the whole territory centering in the visible spectrum and extending to the left to the long Hertzian waves and to the extreme right to the infinitesimally short gamma rays of radium. Nor is it difficult with our minds so attuned to conceive that other portions of this immense elector-magnetic field may discover unsuspected forces as important or even more important than those already known and utilized in therapeutics. Let us therefore approach this subject with open minds.

Remembering that the recognized truths of X-ray and radium were but the scarcely credited marvels of yesterday.

Since we are especially concerned today with the region below but near the violet end of the visible spectrum, I

will refer only to certain demonstrated properties of these so-called Ultra Violet rays.

When the skin is exposed to the rays of the Mercury Quartz Lamp for several minutes at a distance of two feet, a reaction commonly occurs, but only after a latent period of several hours (and in passing it should be noted that the skin reaction, redness and heat, occurs immediately upon exposure to the red or infra-red rays). The usual reaction resembles a moderately sharp sunburn, which in point of fact it virtually is and this reaction lasts several days, according to the degree of burning, to be followed in most patients by pigmentation; compare the "taming" consequent upon repeated sunburning. Should the skin exposed to the Ultra Violet rays be sensitive or the initial exposures prolonged or administered close to the skin, then a blistering occurs, again comparable to that of a severe sunburning. Unlike the inflammatory reaction of X-rays or radium, however, there is no destruction of tissue and consequently no scarring, nor is a Chronic Dermatitis induced, nor Telangiectasis. The worst that may happen, therefore, is a painful burn or a blistering which time and appropriate treatment cures.

I shall later refer to the principles that guide most operators in administering these rays therapeutically and will now proceed to discuss briefly the various beliefs concerning the effect of Ultra Violet radiation.

These are:

A. Local on the skin.
B. General.

A—Local on the Skin:

What is the penetration of the skin by the rays? This is by no means exactly settled, although it is certainly slight and probably not far beyond the superficial layers. The actual depth is likely about one or two millimeters.

*Histologically*, certain changes have been noted: dilatation of the superficial and deep capillaries, loosening and vacuolation of the epidermis, exudation into the corium, a division of the nuclei of the epithelial cells, a migration of the leucocytes and dilatation of the lymph spaces. Repeated exposures lead to pigmentation in most individuals. Next, are the rays Bactericidal?

Experimental data are abundant demonstrating the bactericidal action of Ultra Violet as well as of other electro-magnetic rays. The well-known destructive action of sunlight on bacteria is mainly dependent not upon light *per se* but upon the chemical rays. Their effect is materially intensified and accelerated by moisture and the presence of oxygen.

Certain observations lend weight to an opinion by many and advocated especially by Wichmann that Ultra Violet rays stimulate the production of *anti-bodies* by the skin. Undoubtedly a skin that has been rayed either naturally by sunlight or artificially, becomes more or less immune to infections. For example, during an epidemic of smallpox at Leysin, no patient whose skin had become pigmented as the result of exposure to sunlight, showed any eruption from Variola, whereas the unpigmented suffered severe eruptions.

Again Edgar Mayer reports "that the intradermic tuberculin test in guinea pigs and patients, often gives a reaction of lessened extent when the injected area is treated locally with short exposures of the Ultra Violet rays, either before or after injection. Recently tanned skin gives a slightly lessened reaction."

Further points of interest may be noted:

Ultra Violet rays acting upon Tuberculin for forty-five minutes at a distance of two feet almost completely destroys its power to induce intradermic reactions.

Diphtheria and Tetanus toxin rapidly lose their toxicity whereas their corresponding anti-toxins are much less readily affected. Complement is destroyed.

Vitamines are unaffected (Hess).

Recently an interesting theory has been advanced that Ultra Violet rays break down the cellular elements of the skin, thus releasing cell proteins. These are absorbed and induce a nonspecific immunity response similar to the hypodermic introduction of any foreign protein.

I refrain from offering any opinion on these various observations, but hope that I have impressed upon you as it has been impressed upon me, that *first* the action of the chemical rays upon the skin is associated with profound changes and *second* that these have been earnestly studied by numerous competent investigators.

The *general reaction* is even more interesting and more obscure. The pioneer work of Rollier, amply confirmed by other workers the world over, has demonstrated, at least so far as tuberculosis is concerned, that the healing rays of the sun (believed to be mainly the Ultra Violet rays), exercise a profound influence upon the course of the disease.

In support of this statement I might quote extensively, but will content myself by referring to a paper by Hyde and Lo Grasso of the J. N. Adams Memorial Hospital, Buffalo, which, in particular, care for children suffering from nonpulmonary tuberculosis—bone, joint, glands, etc. Most of their work has been heliotherapeutic, of which they speak with enthusiasm.

''Some of the patients, on admission, present a pitiful picture. They are anemic, emaciated and fever ridden and with features suggestive of suffering; yet, in a few weeks, these patients go through a complete transformation. The pain, often intense, disappears in about ten days; the temperature takes a steady drop, weight is taken on rapidly, the features return to normal and the blood condition is improved. * * * The most characteristic local result that stands out foremost in the treatment of joint tuberculosis by heliotherapy and one of the greatest importance and advantage is, according to Doctor Rollier, the return of motion in the affected joint. He has attained this motion even in cases of fibrous ankylosis where the condition has existed for years. Although we have not had such results in cases of existing fibrous ankylosis, we have attained good motion in early joint tuberculosis * * *."

And elsewhere comment as follows:

* * * "It is well to note that solar radiation is of benefit not only in cases of so-called surgical tuberculosis, but is being applied with excellent results in cases of puerperal sepsis, anemia, convalescence and infectious diseases and in fact in all diseases where the resistance of the patient is below par. It is being used in the European War in the treatment of all kinds of wounds." * * *.

Recently L. F. Hess of New York has published some remarkable observations in connection with Natural and Experimental Rickets and the effect of the Ultra Violet rays on this disorder. In substance he found that rats fed on a rachitic diet (poor calcium Phosphorus and the anti-rachitic vitamine) did not develop rickets if exposed to Ultra Violet radiations, further that the exposure of children suffering from this disease resulted in their cure.

An important contribution to this subject has recently been made by Dr. F. F. Tisdall of this city, working at the Hospital for Sick Children, to this effect, namely, that rachitic children when appropriately exposed to Ultra Violet rays show after a time and during the process of cure an increase in their blood calcium and Phosphorus.

Commenting upon Doctor Hess' address upon this subject before the Royal Society of Medicine in London, Sir William Bayliss stated his belief that in some way actinic rays acting upon the skin, induced the formation of a powerful chemical substance possibly of the nature of a ferment.

Whatever may be the true explanation it appears certain that the rays of the chemical end of the spectrum

(violet, ultra violet and below) exercise a profound influence on metabolism.

So much for certain fundamental scientific observations. Let us now proceed to the practical side of our subject. There are two forms of Mercury Quartz Lamp in ordinary use, namely, the water-cooled lamp (Kromayer) and the air-cooled. The first is used where one desires intense local radiation of short duration, as in the naevus flammeus, and the other both in local radiation of less intensity and where more general radiations are indicated, as in anemia.

The essential parts of both lamps are the same, namely, a vacuum tube of fused quartz containing mercury to which is led a suitable electric current. The circuit is completed by the mercury for a moment or two until the great heat generated, vaporizes it. A brilliant bluish-white light is then emitted which is exceedingly rich in Ultra Violet rays.

Radiation may be either Local or General—local for example in carbuncle, or Lupus Erythematosis or general in anemia or both local and general in Tubercul Ademitis. Where the skin is being rayed the desirable reaction as a rule is one which produces a definite erythema after several hours and lasts a day or two, followed by some pigmentation. The occurrence of pigmentation necessitates continually longer exposures—just as one observes in ordinary exposure to the sunlight along with tanning of the skin.

I will refrain from discussing details of technique further, but perhaps may be permitted to state my belief that it is easily acquired and inasmuch as one can not cause permanent tissue damage or change as with X-ray and radium we may safely proceed to use this method of treatment and learn for ourselves.

The experience of physicians of many years past with the natural energy of the sunlight pointed the way to the use of artificial sources of actinic rays and although it is by no means proved beyond question that the artificially-produced rays are a complete substitute for the natural, still evidence is rapidly accumulating that in the main the therapeutic effect is very similar. Some observers go so far as to favor the artificial, for which it is justly claimed, at least, that it is constantly and immediately available, requires much shorter exposures for apparently similar reactions and can be directed to whatever part of the body accessible to approach from without.

Of course a new, and one believes a powerful form of energy, is bound to be "tried out" in almost every disorder. Extravagant claims based upon enthusiasm and insufficient experience or inaccurate observations or "the pranks of nature" in doing for herself things we ignorantly attribute to our own efforts, are all tending to discount the whole method of treatment. But let me remind you that X-ray and radium therapy and indeed any new method at variance with established principles and practice rightly has been compelled to pass through its "trial by fire." Just because we do not understand or can not comprehend is no justification for ultra-conversatism. You will observe perhaps that I am pleading for the "open mind."

Time will not permit to do much more than enumerate some of the various conditions that have been subjected to treatment by Ultra Violet Rays. Where any comments are offered they are based upon my own experience or upon statements which I, at least, can not question.

LOCAL RADIATIONS

*Dermatological:*

1. Acne, vulgaris and rosacae.
2. Eczema, acute and chronic.
3. Varicose ulcers with eczema.
4. Dermatitis venenata.
5. Carbuncle and furuncle.
6. Certain forms of alopecia (? alopecia areata).

7. Herpes zoster, especially for control of pain.
8. Lichen planus, irritation rapidly controlled.
9. Lupus erythematosis.
10. Lupus vulgaris.
11. Pruritis (vulvae and ani).
12. Psoriasis and seborrheic eczema.
13. Seborrhoea.
14. Burns.
15. Urticaria.
16. ''X''-ray dermatitis and telangiectasis.
17. Icthyosis.
18. Naevus flammeus.

*Nondermatological:*

1. Bruises and hematoma.
2. Gun-shot wounds.
3. Post-operative wound infections.
4. Neuritis, including sciatica and the neuralgias.
5. Myalgia (lumbago, etc.).
6. Teno-synovitis.
7. Gynaecological:
    ? Leucorrhoea.
    ? Pelvic cellulitis, etc.
8. Genito-urinary:
    ? Prostatitis.
    Epididymitis.
    Orchitis.
9. Oto-laryngological:
    Chronic antrum disease.
    Chronic otitis media.
    Tonsillitis.
    Pharyngitis.
10. Diphtheria carriers.

A—General and Local:

1. Osteomyelitis.

2. Most cases of nonpulmonary tuberculosis:
   (a) bone.
   (b) joint.
   (c) Pott's disease.
   (d) discharging sinuses (rectal fistulae).
   (e) laryngitis.

3. All local conditions where the general bodily vigor is low—Asthenic states.

GENERAL RADIATIONS

1. Bronchitis.
2. Anaemia.
3. Psychasthenia.
4. Insomnia.
5. Pulmonary tuberculosis.
6. Chronic sepsis.
7. Tuberculous peritonitis.
8. Genito-urinary tuberculosis.

In conclusion may I quote Edgar Mayor of Saranac Lake:

"Most of our efforts have proved of little avail in teaching us the exact manner of the action of light; and until we know more definitely how it affects protoplasm and the body physiology, we must rely chiefly upon empiricism."

And Bovie:

"When we learn more concerning the relation of the organism and this imponderable part of its environment, radiations will find a place as secure as that now held by chemicals in rational scientific medicine."

